# Vaginal microbiota in women with spontaneous preterm labor versus those with term labor in Kenya: a case control study

**DOI:** 10.1186/s12866-022-02681-0

**Published:** 2022-11-10

**Authors:** Edgar Gulavi, Fridah Mwendwa, David O. Atandi, Patricia O. Okiro, Michael Hall, Robert G. Beiko, Rodney D. Adam

**Affiliations:** 1 Department of Obstetrics and Gynecology, Aga Khan University, Nairobi, Kenya; 2 Department of Pathology, Aga Khan University, Nairobi, Kenya; 3 Faculty of Computer Science and Institute for Comparative Genomics, Dalhousie University, Halifax Nova Scotia, Canada; 4 Department of Medicine, Aga Khan University, Nairobi, Kenya

**Keywords:** Preterm birth, Sub-Saharan Africa, Vaginal microbiota

## Abstract

**Background:**

Preterm birth is a global problem with about 12% of births in sub-Saharan Africa occurring before 37 weeks of gestation. Several studies have explored a potential association between vaginal microbiota and preterm birth, and some have found an association while others have not. We performed a study designed to determine whether there is an association with vaginal microbiota and/or placental microbiota and preterm birth in an African setting.

**Methods:**

Women presenting to the study hospital in labor with a gestational age of 26 to 36 weeks plus six days were prospectively enrolled in a study of the microbiota in preterm labor along with controls matched for age and parity. A vaginal sample was collected at the time of presentation to the hospital in active labor. In addition, a placental sample was collected when available. Libraries were constructed using PCR primers to amplify the V6/V7/V8 variable regions of the 16S rRNA gene, followed by sequencing with an Illumina MiSeq machine and analysis using QIIME2 2022.2.

**Results:**

Forty-nine women presenting with preterm labor and their controls were enrolled in the study of which 23 matched case–control pairs had sufficient sequence data for comparison. Lactobacillus was identified in all subjects, ranging in abundance from < 1% to > 99%, with Lactobacillus iners and Lactobacillus crispatus the most common species. Over half of the vaginal samples contained Gardnerella and/or Prevotella; both species were associated with preterm birth in previous studies. However, we found no significant difference in composition between mothers with preterm and those with full-term deliveries, with both groups showing roughly equal representation of different Lactobacillus species and dysbiosis-associated genera. Placental samples generally had poor DNA recovery, with a mix of probable sequencing artifacts, contamination, and bacteria acquired during passage through the birth canal. However, several placental samples showed strong evidence for the presence of Streptococcus species, which are known to infect the placenta.

**Conclusions:**

The current study showed no association of preterm birth with composition of the vaginal community. It does provide important information on the range of sequence types in African women and supports other data suggesting that women of African ancestry have an increased frequency of non-Lactobacillus types, but without evidence of associated adverse outcomes.

## Background

Preterm birth (PTB) is defined as birth before 37 completed weeks of gestation [1]. It is one of the leading causes of perinatal morbidity and mortality worldwide and about 15 million PTBs occur every year [2]. PTB is a global challenge affecting up to 12% of births in low-income countries and 9% of births in Western countries [3]. The majority of PTBs occur in sub-Saharan Africa and South Asia [4] with an estimate of a 12% PTB rate in sub-Saharan Africa [5]. Kenya has a 12% PTB rate with an estimated 190,000 babies born preterm every year [6].

Maternal–fetal factors and gene–environment interactions play roles in determining the length of gestation. Some of these factors include African ancestry (in the US and the UK), time of less than six months after a previous pregnancy, low prepartum maternal weight, previous preterm birth, multiplex pregnancy, and maternal infection or vaginal dysbiosis, as well as numerous other known or suspected risk factors [7, 8]. The vaginal microbiota is thought to play a role in pregnancy outcomes. In addition, vaginal dysbiosis has been associated with preterm labor [9]. Since African American women are at greater risk for vaginal dysbiosis and PTB than white women [9], it is important to understand any difference in vaginal microbiota of women of African vs. European ancestry. Ravel et al. [10] used 16S rRNA gene sequencing to analyze 98 vaginal swabs from European women and 104 vaginal swabs from African American women and classified the corresponding samples into five major groups termed *Community State Types* (CST). Four CSTs have predominantly *Lactobacillus*, including CST I (*Lactobacillus crispatus*), CST II (*Lactobacillus gasseri*), CST III (*Lactobacillus iners*), and CST V (*Lactobacillus jensenii*). CST IV comprises strict anaerobes that are often associated with bacterial vaginosis (BV) such as *Prevotella, Gardnerella, Sneathia* and *Atopobium* species. In that study, CST I was the most common CST among European women while CST IV was the most common in African American women [10]. Other studies have also shown that CST IV is more common in women of African descent than those of European descent [11, 12]. However, one study showed that the difference between white and black women disappeared when women with evidence of BV by Nugent’s criteria were excluded [13].

Attempts to determine associations between PTB and specific CST types or other designations of vaginal microbiota have also produced differing results. These studies of women with PTB have shown an association of PTB for Caucasian women with an increased Shannon Diversity Index [14], no correlation between CST and PTB in African American women [2], or an association of CST IV (*Lactobacillus*-poor) with PTB that was more pronounced with the presence of *Gardnerella* or *Ureaplasma* [15]. A recently reported meta-analysis using sequence data from five studies [2, 14–17] found that the vaginal microbiota from women with preterm delivery showed greater within-sample variation than those with term delivery and was found across racial groups [8]. They also found that three genera; *Gardnerella*, *Lactobacillus*, and *Aerococcus* were associated with third trimester preterm birth.

Data available at the time of initiating the study suggested the presence of a distinct placental microbiota [18], and also raised the question of whether there was an association between placental microbiota and the occurrence of PTB.

A better comprehension of the changes in vaginal microbiota during pregnancy could pave the way to predictive diagnostics and focused treatments of the complications associated with the intricate process of pregnancy, labor and birth. In the current study, we used a cohort study to determine whether there was a difference in the microbiota of women presenting with preterm labor compared to full term. In addition, we analyzed the placental microbiota to investigate any potential associations with preterm labor.

## Methods

### Study site

Aga Khan University Hospital (AKUH) is a 280-bed teaching hospital in Nairobi, Kenya that is accredited by the US-based Joint Commission International and has a full range of obstetric and neonatal services. Approximately 3600 deliveries per year are performed.

### Participant recruitment

Pregnant women over the age of 18 years presenting in active labor or with preterm pre-labor rupture of membranes (PPROM) between 26 and 36 6/7 weeks gestation were recruited into the study from March 2018 to March 2019. Patients were excluded if they had medically indicated preterm delivery (for example preeclampsia, intrauterine growth restriction or congenital anomalies), antibiotics given more than 24 h prior to enrollment or within the last 4 weeks, cervical cerclage, progesterone supplementation, or HIV infection.

A control group of mothers matching the study group as closely as possible for age and parity but presenting in labor at term (37 completed weeks) were enrolled in a 1:1 ratio. We considered a pregnancy to be normal if there were no obstetric or medical complications. Comparisons between the cases (preterm) and controls (term) were made using the Chi-square test and for nonparametric data, the Mann–Whitney test was used.

### Sample collection and analysis

A physician or midwife collected the vaginal samples under direct visualization by swabbing the posterior vaginal fornix 3 to 5 times using sterile Snappable Polystyrene & Viscose Amies Swabs (Deltalab, Barcelona, Spain). Samples were stored at -80 ˚C until testing. Genomic DNA extraction was carried out using QIAamp DNA Mini Kit (Qiagen, Germany) as per manufacturer’s protocol.

The placenta was collected into a clean ziplock bag after delivery and immediately transferred to a dedicated 4 °C refrigerator. Aseptically, a placental sample was cut from both fetal and maternal internal structures to minimize the risk of surface contamination. The samples were transferred to a -80 °C freezer for storage until DNA extraction. DNA extraction was carried out using Dneasy Blood & Tissue Kit (Qiagen, Germany) as per manufacturer’s protocol.

Extracted DNA samples were shipped to the Dalhousie Integrated Microbiome Resource (IMR, Halifax, Nova Scotia, Canada) for sequencing. The protocol for sequencing is described at https://imr.bio/protocols.html#library; in brief, extracted DNA was amplified using PCR, targeting the conserved 16S ribosomal RNA gene. PCR primers amplified the V6/V7/V8 variable regions of the gene, providing over 400 nucleotides to use for species identification. Amplified DNA libraries were sequenced using an Illumina MiSeq machine. Sequencing runs were stored as FASTQ files; vaginal files with at least 2,000 associated reads were retained for subsequent analysis, while the minimum threshold for inclusion of placental samples was 100 reads.

### Microbial CST analysis

Downstream analysis of DNA sequence data was performed using QIIME2 2022.2 [19]. Sequences were denoised using DADA2 [16] version 2022.2.0, with left and right truncation lengths of 280 and 270 nt, respectively. Primers were trimmed in both directions. Taxonomic assignment was performed as follows: reads were classified with the Naïve Bayes classifier using the SILVA version 138 reference database, with a minimum confidence score of 0.7 required to make a classification at a given taxonomic level. Taxonomic distributions were visualized using the “barplot” command of the “taxa” plugin. Community state assignments were based on the dominant *Lactobacillus* species for CST I (*L. crispatus*), II (*L. gasseri*), III (*L. iners*), and V (*L. jensenii*); samples that were dominated BV-associated taxa such as *Prevotella, Gardnerella, Sneathia* and *Atopobium* were assigned to CST IV. Tests for significant differences for the control vs. pre-term cohorts were performed using ALDEx2 [20], which addresses the issue of compositionality using the centered log-ratio transformation. Effect sizes and *p*-values were calculated using the QIIME2 “q2-aldex2” plugin’s “effect_plot” command, which computes both the Welch’s t-test and Wilcoxon test with Benjamini–Hochberg correction for multiple hypotheses. Alpha diversity values were computed for all samples using the Faith’s phylogenetic diversity and Shannon entropy measures, rarefaction curves were generated, and group differences between case and control samples were tested by the nonparametric Kruskal–Wallis test.

### Ethical considerations

Ethics approval was obtained from the AKUH Ethics Review committee (2017/REC-86). Samples were collected only once during routine evaluation of women presenting with labor. For women in early labor, a written consent was followed by sample collection. For those in active labor, verbal assent was sought during labor for the sample collection; then written consent was sought after delivery. If the written consent was denied, the samples were discarded.

## Results

### Baseline characteristics

**Fig. 1:**
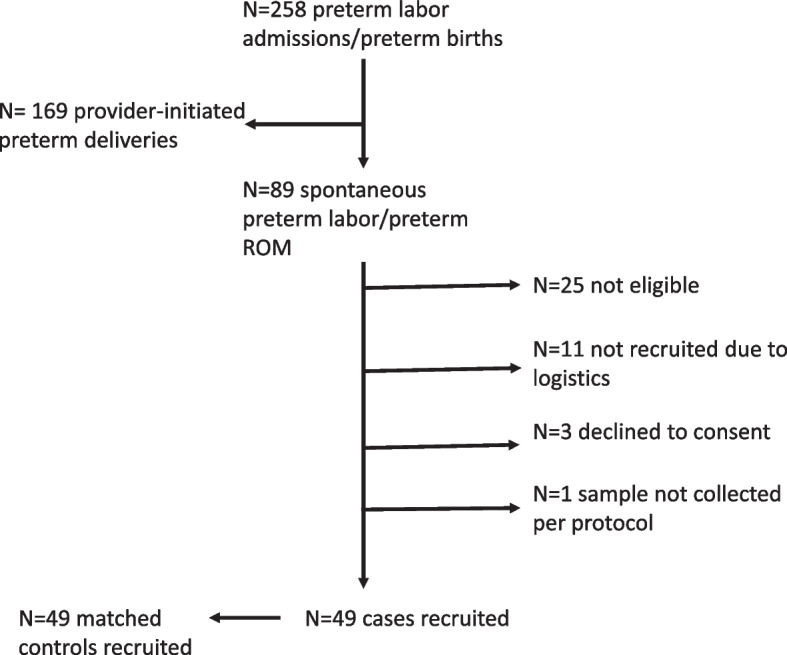
Study flow chart showing recruitment of participants

A total of 98 patients were recruited for the study between March 2018 and March 2019. Of these, 49 were patients with preterm labor who met the criteria and 49 were matched term controls (Fig. 1). The mean age of the participants was 32.2 with 24 years being the minimum age and 44 years as the maximum with no significant differences between the case and control groups (Table 1). There were 44 (89.8%) Africans, three (6.1%) Caucasians and two (4.1%) East Asians in the preterm group, and 48 (98%) Africans and one Caucasian in the control group. Most patients were nonvegetarian (*n* = 94, 95.9%) and did not have a history of prior PTB (*n* = 86, 87.7%). The majority of the controls (*n* = 45, 91.8%) were delivered vaginally, in comparison with only 18 (36.7%) in the preterm group. In addition, there was a significant difference in the gestational age by days between the two groups. The preterm group had a mean gestational age of 224.6 while the term group had a mean of 276.9 (Table 1).

**Table 1:** Social and demographic characteristics of the preterm and term groups

CHARACTERISTIC	CASES (Preterm)* N* = 49 (%)	CONTROLS (Term)* N* = 49 (%)	*P* VALUE
**Maternal Age** (Mean)	32.7	31.5	0.285 z-score = -1.0664
	32.7 ± 5.10	31.5 ± 4.50	0.25^a^
**Ethnicity**			
African	44(89.8%)	48 (98%)	0.52^b^
Caucasian	3 (6.1%)	1 (2%)	
East Asian	2 (4.1%)	0 (0%)	
**Marital Status**			
Married	41 (83.7%)	39 (79.6%)	0.60^b^
Single	8 (16.3%)	10 (20.4%)	
**Highest education attained**			
Tertiary	40 (81.6%)	43 (87.5%)	0.54^b^
Secondary	8 (16.3%)	6 (12.8%)	
Primary	1 (2%)	0 (0%)	
**Diet**			
Nonvegetarian	46 (93.9%)	48 (98%)	0.30^b^
Vegetarian	3 (6.1%)	1 (2%)	
**Parity**			
Primigravida	12 (24.5%)	32 (65.3%)	0.0007^b^
1 -2	23 (47%)	12 (24.5%)	
3–4	12 (24.5%)	4 (8.2%)	
5 or More	2 (4.1%)	1 (2%)	
**Previous Preterm Delivery**			
Present	10 (20.4%)	2 (4.1%)	0.01^b^
Absent	39 (79.6%)	47 (95.9%)	
**Mode of delivery**			
Elective Caesarean Section	4 (8.2%)	1 (2%)	< 0.00001 ^b^
Emergency Caesarean Section	13 (26.5%)	3 (6.1%)	
Spontaneous Vaginal Delivery	18 (36.7%)	45 (91.8%)	
Unknown^c^	14 (28.6%)	0 (0%)	
	Mean ± SD		
**Gestational age (Mean days)**	**224.6**	**276.9**	** < 0.00001 **^**a**^ **z-score = 8.657**

^a^Mann-Whitney test for nonparametric data

^b^Chi-square test

^c^Women who transferred out before delivery grouping of vaginal samples in Fig. 4)

### DNA sequence analysis

**Fig. 2:**
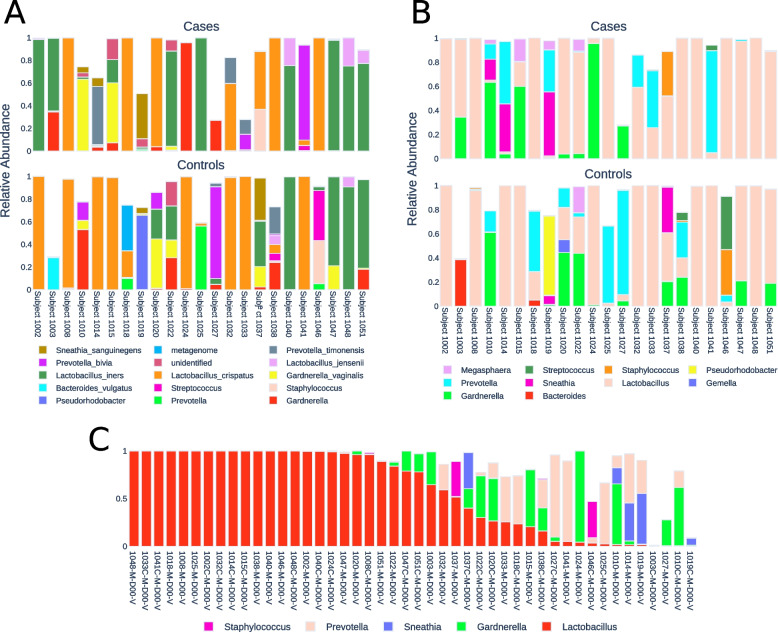
Relative abundance of 16S reads assigned to **a** the 15 most abundant species-level designations and **b** the 15 most common genera of the 23 matched case–control vaginal samples. **c** Case (pre-term) and control (full term, designated with C) samples sorted in decreasing order of *Lactobacillus* abundance. Assigned taxa not in the top 15 for each rank were assigned to a uniform “Other” category and have no assigned color

A total of 100 vaginal and 71 placental samples were sequenced from the mothers in the case and control groups. The average read count was 181 and 26,798 per sample for placental and vaginal samples, respectively, after primer trimming, quality filtering, overlap assembly, and chimera removal. Vaginal samples with fewer than 2,000 reads were excluded from downstream analysis, leaving 74 vaginal samples (average of 35,932 reads per sample, total of 2,658,997 reads). Rarefaction curves of the vaginal samples suggest sequencing depth was sufficient to capture the majority of abundant taxa (data not shown), and a test of group differences revealed no significant difference in alpha diversity between case and control vaginal samples (Fig. 2). A total of 13 placental samples had read counts > 100 and were retained in the final data set.

### Composition of the vaginal microbiota

**Fig. 3:**
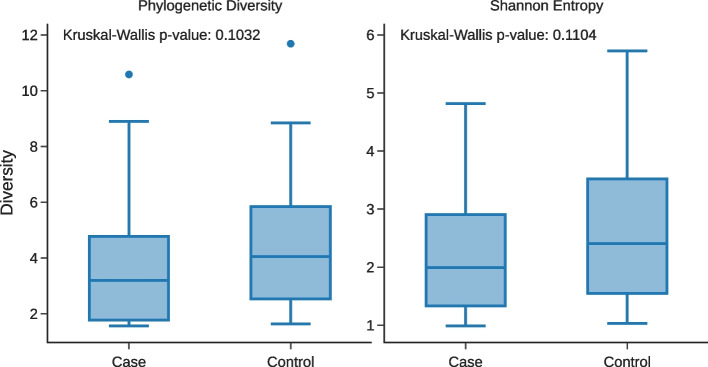
Comparison of alpha diversity measurements of cases with PTB and controls with term delivery. Boxplot shows the quartiles and whiskers extend to 1.5 times the inter-quartile range

**Fig. 4:**
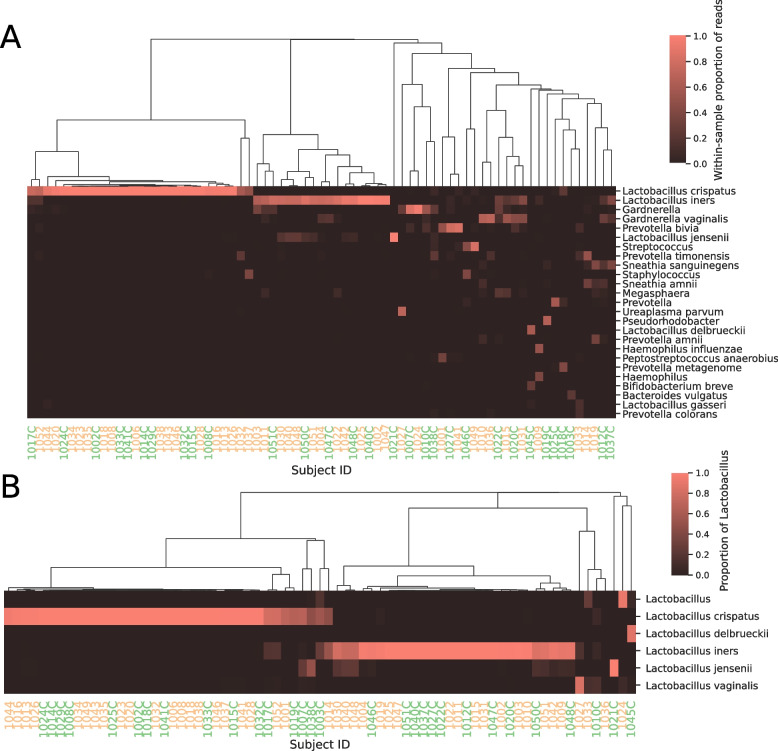
Heatmaps of **A**) the proportion of reads from the top 25 taxonomic classifications across all vaginal samples and **B**) the proportion of reads from the top 5 species across all vaginal samples belonging to the *Lactobacillus* genus. Genus names indicate reads that are classified at the genus level but without a confident classification at species level. Hierarchical clustering dendrograms for samples were computed using complete linkage. “**C**” indicates full term control sample

Based on the SILVA taxonomic classification we observed three distinct species of *Lactobacillus* with an average abundance > 0.1% across all 74 high recovery vaginal samples: *L. iners* (43 individuals, 22.3% relative abundance)*, L. crispatus* (50 individuals, 37.5% relative abundance)*,* and *L. jensenii* (20 individuals, 0.3% relative abundance). An additional six named species were observed in lower abundance, most notably *L. vaginalis* which was found in 34 individuals but with an average abundance of only 0.16% (Fig. 3a and 4). Vaginal samples with *L. crispatus* tended to contain no other named species of *Lactobacillus*, while *L. iners* was found either alone or in association with *L. jensenii* as a minor component of the sample (Fig. 4). No amplicon sequence variants (ASVs) had a differential abundance that was significant between pre-term and term birth according to ALDEx2; the smallest Benjamini–Hochberg corrected *p*-value was 0.843.

At the genus level, 73 out of 74 vaginal samples contained at least a small number of reads that were assigned to *Lactobacillus*, with a mean of 64.4% across all samples (Fig. 3b-c). Vaginal samples not dominated by *Lactobacillus* (such as subject 1036 with zero *Lactobacillus* reads; Fig. 4b) were generally dominated by genera commonly associated with BV (Fig. 4). *Gardnerella* and *Prevotella* were each found in 39 and 50 samples, respectively, with an average abundance of 11.1% and 8.8%. Other genera were found in relatively few samples, although often with high abundance: for example, *Sneathia* had a maximum abundance of 53.05% across 15 samples, while *Pseudorhodobacter* was present in only two samples but with an abundance of 65.49% in one sample. Conversely, several genera were found in many samples but at consistently low levels, including *Dialister* (33 samples; max abundance = 6.24%), *Corynebacterium* (25 samples, max abundance = 2.64%), and *Atopobium* (22 samples, max abundance = 8.77%). *Streptococcus* was found in 21 samples (10 case, 11 control) with an average abundance of 1.9% and a maximum of 82.75%.

Of the 23 case–control pairs, all fell into three of the originally described CSTs [10], types I, III and IV (Table 2). There was no clear difference between the case and control groups in their CST assignment. When all 73 of the sequenced specimens were included (43 preterm and 30 term) whether or not they were matched, the results were similar with the same three CSTs dominating (Table 2). In this larger group, there were 28 individuals that could be considered as a part of CST I (*L. crispatus*), 18 pre-term birth cases and 10 controls; 17 individuals associated with CST III (*L. iners*), 12 pre-term birth cases and 5 controls; 1 individual associated with CST V (*L. jensenii*), a control; and 27 individuals associated with CST IV, 13 pre-term birth cases and 14 controls (Fig. 3). In this study’s cohort, *L. crispatus* was not associated with term birth and, conversely, a significant number of the cases and controls had a predominance of *Gardnerella* and/or *Prevotella*, but no association with preterm labor (demonstrated by the lack of significantly differentially abundant ASVs and the mixture of cases and controls in each.

**Table 2:** Community Sequence Types (CST)

CST Number	Description	Cases with sequences from matched controls (23 in each group)		All cases and controls with sequences (43 and 30)	
		Case (Preterm)	Control (Term)	Case (Preterm)	Control (Term)
I	*L. crispatus*	7	8	18	10
II	*L. gasseri*	0	0	0	0
III	*L. iners*	8	4	12	5
IV	Diverse	8	11^1^	13	14
V	*L. jensenii*	0	0	0	1

The designation of Ravel et al. is used [10]

^1^Two of the control participants designated as CST IV had co-existence of *G. vaginalis* and *L. iners*

### Placental samples

**Fig. 5:**
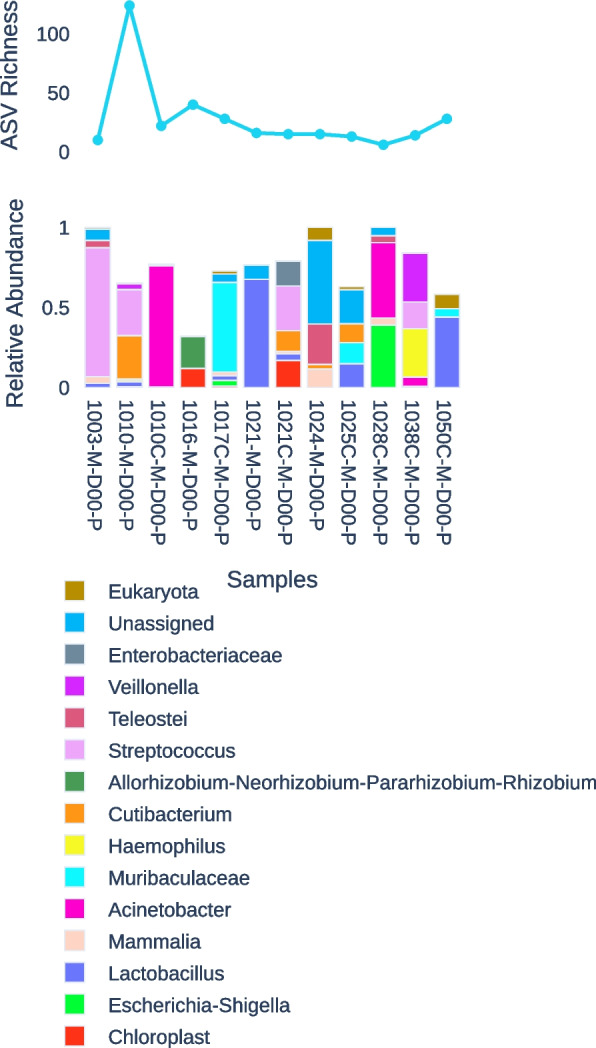
Raw read counts (top) and relative abundance (bottom) of reads assigned to the most-common taxonomic groups of genus-level designations in 13 placental samples with passed read count > 100

Of the 71 placental samples (Fig. 5), only 13 (17.6%) had more than 100 reads that passed the quality threshold, and only two had more than 2,000. The 13 samples split nearly evenly between cases (5/13) and controls (8/13). Many samples with fewer than 100 sequences were dominated either by poorly classified reads that mapped only to “Bacteria” or “Phylum OD1”. Forty-four samples had at least one sequence that was classified at a lower taxonomic rank; twelve of these had classified reads that mapped only to *Lactobacillus*. *Lactobacillus* was not identified in an additional twelve samples.

However, five placental samples showed evidence of *Streptococcus* with abundance between 4.4% and 80.5%; *Streptococcus agalactiae* was identified in a previous study as the only species that could be confidently recovered [21]. The sample with the highest percentage of *Streptococcus* (Case 1003) had a corresponding vaginal abundance of 0.78%; a rectal swab taken from the neonate immediately after birth yielded 99.6% *Streptococcus*, with the remaining sequence reads assigned to *Enterobacteriaceae.*

## Discussion

The present study is the first gene sequencing‐based vaginal microbiota study to date in Kenya with a case–control design comparing the vaginal microbiota of between women with spontaneous preterm labor with those who went to full term. The major objective was to identify whether there were any vaginal microbiota patterns associated with preterm labor in the Kenya population. We found no difference in CSTs between cases with preterm labor and controls with term labor.

Similar to other studies of the vaginal microbiota, we observed vaginal microbial communities with a high incidence of species within the genus *Lactobacillus*; however, the number of distinct species groupings according to our ASV analysis was small, with the predominant species identified being *L. iners* and *L. crispatus* (Fig. 5). The ecological significance of these associations is unclear, and future metagenomic analysis may yield insights into the patterns we describe here. Although *Lactobacillus* was widely distributed across subjects as expected, a substantial number of both case and control samples had substantial counts of other genera, with 20/46 paired samples having a *Lactobacillus* relative abundance < 50%. These samples were dominated by bacteria such as *Prevotella* and *Gardnerella* that are frequently associated with BV. BV has long been associated with PTB and treatment with metronidazole has been used to prevent PTB [22]. However, treatment of asymptomatic BV did not reduce PTBs [23]. Thus, it is of interest to determine whether any of the five CSTs or individual organisms are associated with PTB, especially for CST IV or *Gardnerella*. Indeed, distinct taxa have been associated with PTB in a number of studies. In support of this possibility, a study of Indian women showed that *L. iners*, *Megasphaera*, *G. vaginalis*, and *Sneathia sanguinegens* were higher in women presenting in preterm labor, while *L. gasseri* was higher in those presenting at term [24]. In addition, *L. crispatus* has been protective in other studies [25] and the suggestion that *L. crispatus* is incompatible with *G. vaginalis* has supported the idea of a protective effect of *L. crispatus* [26]. A study of vaginal metabolites and preterm labor in the setting of a mostly white population of British women also suggested a protective effect of *L. crispatus* and an association of preterm labor with *L. jensenii* [27], while the Peruvian study noted above showed no association [28]. Some studies of women of African ancestry have found an association of PTB with certain taxa [29], while others have not [2, 17]. In addition, there is evidence for an increased frequency of non-*Lactobacillus*-related CSTs in women of African ancestry, but not necessarily associated with adverse outcomes [30]. Other microbial associations have also been described, including a study of Korean women that showed an association of *Klebsiella* in the vaginal microbiota and preterm labor [31]. *Klebsiella* is part of the *Enterobacteriaceae* class, which was not associated with preterm labor in our study. In the current study, nearly all the women fell into CST I, III, or IV, but the proportions of preterm and term did not show major differences for these three types. Our study also found no trend for an association with the presence of sequences from the genera *Lactobacillus*, *Gardnerella*, and *Aerococcus* that were associated with third semester PTB in a meta-analysis [8]. However, it is possible that certain relevant associations were missed in the current study in view of the relatively small number of matched cases and controls. In addition to the lack of difference of CST for preterm vs. term delivery, there was also no difference between the two groups for measures of alpha diversity.

Our study also addressed the question of whether a placental microbiome is associated with PTB. This is especially important since some studies have suggested a placental microbiome or an association with certain outcomes [18], while other studies have found no evidence of a specific placental microbiome [32, 33]. In our study, the read counts and taxonomic affiliations of our placental samples were largely consistent with very low bacterial loads that have been reported elsewhere in the literature [34]. Many samples either returned no reads or were dominated by common vaginal flora (most notably *Lactobacillus*), unclassified sequences, or eukaryotic sequences and the *Bradyrhizobium* group that are likely contaminants. The very low read recovery in many placental samples may reflect difficulty in recovering viable DNA samples from placental matter. However, the presence of organisms including *Lactobacillus* and *Veillonella* suggests the more likely explanation that many “placental” samples are dominated by bacteria acquired during passage through the birth canal. These observations are consistent with reports that suggest placentally derived bacteria (i.e., a “placental microbiome”) are rare [32–36]. Thus, our observations are consistent with the evidence that there is not normally a separate placental microbiota. However, the few placental samples with the highest sequence recovery were often dominated by *Streptococcus*, which is interesting in light of the common association of *S. agalactiae* (Group B streptococci) with adverse maternal and neonatal outcomes [37].

## Conclusion

In summary, these results contribute to the increasing data that shows that there is a spectrum of diversity in the vaginal microbiota without clear evidence of specific microbiota types that have a correlation with preterm labor. Therefore, an understanding of the variables associated with African ethnicity that contribute to this diverse microbiota has important implications regarding reproductive health outcomes.

This study is a fundamental step towards gathering more information on the relationship of vaginal microbiota and PTB which would help us to establish a greater degree of accuracy on future implications of this portentous relationship.

## Data Availability

“Sequence data are available at the European Bioinformatics Institute at project accession PRJEB48940. The name of the database is “BMCMicrobiology Preterm Birth.’ Analysis scripts can be accessed at https://github.com/mwhall/BMCMicrobiology_Preterm_Birth”.

## Abbreviations

PTB: Preterm birth
CST: Community state types
BV: Bacterial vaginosis
Rrna: Ribosomal ribonucleic acid
AKUH: Aga Khan University Hospital
PPROM: Preterm pre-labor rupture of membranes
PCR: Polymerase chain reaction
Nt: Nucleotides
ASV: Amplicon sequence variant
